# Advances in tumor necrosis factor-α inhibitors for the treatment of juvenile idiopathic arthritis-associated uveitis

**DOI:** 10.3389/fimmu.2026.1966054

**Published:** 2026-09-07

**Authors:** Hanbing Ding, Songtao Fan, Xiang Ma

**Affiliations:** Department of Ophthalmology, The First Affiliated Hospital of Dalian Medical University, Dalian, China

**Keywords:** adalimumab, anti-drug antibodies, juvenile idiopathic arthritis associated uveitis, therapeutic drug monitoring, treatment tapering, tumor necrosis factor-α inhibitors

## Abstract

Juvenile idiopathic arthritis-associated uveitis (JIA-U) is a common and sight-threatening extra-articular manifestation of juvenile idiopathic arthritis, typically presenting as chronic noninfectious anterior uveitis. Its early symptoms are often insidious, and delayed detection and treatment may lead to cataract, glaucoma, macular edema, and irreversible visual impairment. Conventional treatment is based on glucocorticoids and immunosuppressants; however, some children continue to experience inadequate efficacy, glucocorticoid dependence, drug intolerance, or adverse events. In recent years, substantial progress has been made in the use of biologics for JIA-U. Among them, tumor necrosis factor-α inhibitors, particularly adalimumab, have shown potential for controlling ocular inflammation, reducing glucocorticoid exposure, and improving visual outcomes, thereby offering a new therapeutic avenue for patients with JIA-U. Although TNF-α inhibitors have become an important component of systemic therapy for JIA-U, optimal dosing, long-term maintenance, tapering, and the timing of discontinuation require further investigation. Additional prospective, disease-specific pediatric studies are needed to define the optimal timing of TNF-α inhibitor initiation, precise dose-adjustment strategies, and long-term safety management, thereby advancing individualized and precision treatment for JIA-U.

## 1 Introduction

Juvenile idiopathic arthritis-associated uveitis (JIA-U) is one of the most common and sight-threatening extra-articular manifestations of juvenile idiopathic arthritis (JIA) (1). It typically presents as chronic nongranulomatous anterior uveitis with an insidious onset (2). If not promptly detected and treated, some patients may develop complications such as cataract, glaucoma, band keratopathy, and macular edema; severe cases may result in irreversible visual loss or even blindness (3). Glucocorticoids can rapidly suppress ocular inflammation, but prolonged use increases the risk of ocular complications such as cataract and glaucoma and is therefore unsuitable for long-term maintenance therapy. Methotrexate (MTX) is the most commonly used first-line systemic immunomodulatory agent and can reduce intraocular inflammatory activity and glucocorticoid dependence; nevertheless, some children have an inadequate response, drug intolerance, or adverse events (4). The long-term management of JIA-U remains challenging, underscoring the need for more effective and safer therapeutic strategies.

In recent years, the rapid development of biologics has expanded the therapeutic options for JIA-U. Current guidelines recommend preferential use of monoclonal antibody inhibitors of tumor necrosis factor-α (TNF-α), particularly adalimumab and infliximab, in patients with persistent ocular inflammation despite conventional systemic therapy (4, 5). TNF-α inhibitors currently used in clinical practice include adalimumab (ADA), infliximab (IFX), golimumab (GLM), certolizumab pegol (CZP), and etanercept (ETN) (6). Although these agents have improved inflammatory control and visual outcomes in some patients, a subset of children continue to have persistent articular or ocular inflammation, and available therapies do not invariably achieve sustained disease remission (7). Previous studies have focused primarily on therapeutic efficacy, whereas several clinically important issues remain unresolved, including inadequate response to standard ADA dosing, the optimal timing of treatment intensification or switching, and strategies for maintenance, tapering, and discontinuation. Accordingly, this review aims to comprehensively assess the efficacy, safety, drug selection, dose optimization, and tapering or discontinuation strategies of TNF-α inhibitors in JIA-U to support individualized clinical management.

## 2 Materials and methods

### 2.1 Review approach and evidence selection

This narrative Mini Review synthesizes randomized trials, systematic reviews, guidelines, cohort and retrospective studies, and case reports cited herein. Evidence on TNF-α inhibitor efficacy, safety, selection, dose optimization, therapeutic drug monitoring, and tapering in JIA-U was prioritized; heterogeneous noninfectious uveitis studies were included when JIA-U-specific evidence was limited.

### 2.2 Diagnostic framework and statistical reporting

No patients were enrolled and no study-specific diagnoses were made. Diagnostic information was interpreted within established JIA and ophthalmic assessment frameworks, including slit-lamp evaluation and exclusion of infectious or alternative inflammatory causes. No new meta-analysis or inferential testing was performed; numerical results were reported descriptively as presented in the source studies.

## 3 Results of the narrative evidence synthesis

### 3.1 TNF-α inhibitors

TNF-α is an important proinflammatory cytokine produced by macrophages, T lymphocytes, natural killer cells, and other cell types. Its excessive activation contributes to chronic inflammation in autoimmune diseases (8). In intraocular inflammation, TNF-α acts through tumor necrosis factor receptor 1 (TNFR1) and tumor necrosis factor receptor 2 (TNFR2) expressed on iris, ciliary body, and retinal pigment epithelial cells and promotes vascular endothelial growth factor production by choroidal endothelial cells, thereby contributing to uveitic macular edema and tissue injury (9). TNF-α promotes persistent intraocular inflammation and tissue damage, whereas targeted TNF-α inhibition can attenuate the immune-inflammatory response, control ocular inflammation, and reduce the risk of visual impairment (10). As understanding of the immunoinflammatory mechanisms underlying JIA-U has advanced, TNF-α inhibitors have emerged as an important therapeutic option for refractory disease.

### 3.2 Adalimumab

ADA is a fully human monoclonal antibody against TNF-α (11) and plays an important role in the treatment of JIA-U. The recommended dose in clinical practice is 20 mg subcutaneously every 2 weeks for patients weighing <30 kg and 40 mg subcutaneously every 2 weeks for those weighing ≥30 kg (12).

Current evidence supports the favorable efficacy and safety of ADA as TNF-α inhibitor therapy for JIA-U (13, 14). ADA can effectively control ocular inflammation, reduce glucocorticoid dependence, and improve visual outcomes (15). A systematic review by Renton et al. included three randomized controlled trials involving 134 children with JIA-U aged 2–18 years. ADA increased the likelihood of treatment success and reduced the use of topical glucocorticoids, supporting its consideration as a preferred TNF-α inhibitor for JIA-U (16). The randomized, double-blind SYCAMORE trial was stopped early for efficacy after 90 participants had been randomized. Treatment failure occurred in 16 of 60 patients (27%) receiving adalimumab plus methotrexate, compared with 18 of 30 patients (60%) receiving placebo plus methotrexate (hazard ratio, 0.25; 95% confidence interval, 0.12–0.49; p < 0.0001). However, adverse-event rates were higher in the adalimumab group than in the placebo group (10.07 vs. 6.51 events per patient-year), emphasizing the need to balance efficacy against treatment-related risks (12). Schmeling et al. reported that ADA was generally effective and had an acceptable safety profile in JIA, with greater efficacy when used as the first biologic. Nevertheless, uveitis flares still occurred in some patients, suggesting that ocular inflammatory activity may be independent of the progression of articular disease and underscoring the need for continuous ophthalmic monitoring (17). In addition, Işık et al. retrospectively screened 2,385 patients with JIA and identified 101 cases of new-onset uveitis. Among 78 patients with uveitis treated with ADA, 64 achieved remission, corresponding to a remission rate of 82.1%. The remission rate after switching from ETN to ADA was 70.3%, and no new-onset uveitis was observed in the ADA-treated group. These findings suggest favorable control of intraocular inflammation, although prospective studies are still required for confirmation (18).

Current evidence suggests that ADA should be considered one of the preferred biologics when MTX is ineffective or not tolerated (19). Although ADA is the most widely used and best-supported TNF-α inhibitor for JIA-U, some patients exhibit primary nonresponse or secondary loss of response. Whether ADA should be continued in patients with an inadequate response or secondary loss of response to standard biweekly dosing remains controversial.

### 3.3 Infliximab

IFX is a human-murine chimeric monoclonal antibody administered by intravenous infusion. A commonly used induction regimen consists of infusions at weeks 0, 2, and 6, followed by maintenance infusions every 4–8 weeks according to disease activity and treatment response; the recommended dose is generally 5–10 mg/kg per infusion (5).

Available studies indicate that IFX provides effective inflammatory control, preserves visual acuity, and facilitates glucocorticoid tapering in JIA-U. An analysis by Xiong et al. that included 16 observational studies showed that IFX achieved a high rate of intraocular inflammation control in refractory noninfectious uveitis, helped maintain or improve visual acuity, and reduced glucocorticoid use. However, the included etiologies comprised JIA, Behçet disease, sarcoidosis, and spondyloarthritis-associated uveitis, and the findings therefore constitute indirect evidence for JIA-U (20). Kreps et al. reported that switching from ADA to IFX enabled a substantial proportion of patients with JIA-U to achieve inflammatory control, suggesting that IFX is an important subsequent option after an inadequate response or secondary loss of response to ADA (21). For patients with an inadequate response to the standard regimen, dose escalation may be considered under close monitoring. Sukumaran et al. retrospectively analyzed 34 children with refractory uveitis who received IFX for more than 1 year. After 6 months of treatment, intraocular inflammation had decreased in most children, and topical glucocorticoids could be tapered or discontinued. By the end of the study, 82.4% of the children required IFX at ≥10 mg/kg, suggesting that higher doses or shorter dosing intervals may improve inflammatory control in some patients who respond inadequately to standard dosing (22). However, the optimal dose for pediatric JIA-U has not been established.

Regarding the comparative efficacy of IFX and ADA, a study by Cecchin et al. on the medium- to long-term management of JIA-U further indicated that both agents reduced uveitis recurrence, whereas ADA may be associated with higher rates of clinical remission and drug retention as well as a more favorable safety profile (23). Thus, given the current strength of evidence and guideline recommendations, ADA remains the more commonly used first-line TNF-α inhibitor for JIA-U, whereas IFX is better suited as an alternative for patients with severe or recurrent disease or an inadequate response to ADA. Compared with ADA, however, evidence regarding IFX in JIA-U is relatively limited; most available studies enrolled heterogeneous populations with noninfectious uveitis, and disease-specific evidence in children with JIA-U remains insufficient. Additional prospective studies in pediatric JIA-U populations are needed to define the optimal IFX dose, dosing interval, combination regimen, and long-term safety.

### 3.4 Golimumab

GLM is a fully human monoclonal antibody against TNF-α (24). It is available in subcutaneous (SC) and intravenous (IV) formulations, although its ocular use remains off-label and no head-to-head comparison between the two routes has been conducted in JIA-U. SC administration is more convenient for outpatient or home-based treatment, whereas IV administration provides supervised, body-surface-area-based dosing at longer maintenance intervals but requires infusion-center resources. Lanz et al. reported 10 patients with JIA-U who received subcutaneous GLM after an inadequate response to ADA. All eight patients who switched to GLM because of secondary loss of response initially responded; neutralizing anti-adalimumab antibodies (AAA) were detected in five, although three subsequently lost response during follow-up. The two patients with primary nonresponse to ADA did not improve after switching to GLM (25). These findings suggest that within-class switching may be more appropriate for immunogenicity-related or secondary failure and may provide limited benefit in primary nonresponse. A systematic review and meta-analysis by Okada et al. suggested that golimumab (GLM) may provide both inflammation control and corticosteroid-sparing benefits in refractory noninfectious uveitis, although all included regimens were SC and the certainty of the evidence was low (26). In an IV series reported by Khan et al., 11 of 13 patients with JIA-associated anterior uveitis (84.6%) responded, and cystoid macular edema resolved in all six affected eyes. These response rates should not be directly compared because of the small sample sizes, different baseline characteristics, concomitant treatments, and nonuniform response definitions (27).

### 3.5 Certolizumab pegol

CZP is a pegylated Fab′ fragment that inhibits TNF-α and lacks a fragment crystallizable (Fc) region (28). A case study by Sharon and Chu showed that CZP improved some cases of refractory noninfectious uveitis without apparent adverse events; however, its efficacy in the included case of JIA-U was limited, and IFX subsequently had to be reintroduced (29). Evidence is somewhat broader for GLM than for CZP, but neither agent has randomized JIA-U-specific support. Larger studies, longer follow-up, and disease-specific pediatric investigations are needed to clarify the efficacy and long-term safety of GLM and CZP in JIA-U.

### 3.6 Etanercept

ETN effectively controls articular disease in many patients with JIA, but its efficacy against ocular inflammation is inconsistent. Armaroli et al. reported an 18-year clinical study showing stable long-term control of articular disease in JIA with ETN, but evidence supporting its efficacy against ocular inflammation in JIA-U was lacking (30). In addition, new-onset or paradoxical uveitis may occur during ETN exposure, and the underlying mechanism remains incompletely understood (31). Overall, the value of ETN in JIA lies primarily in controlling articular disease rather than ocular inflammation. It should not be considered a preferred TNF-α inhibitor for JIA-U, and regular ophthalmic screening remains necessary during treatment. The comparative evidence, reported ocular outcomes, and clinical positioning of these agents are summarized in **Table 1**.

**Table 1:** Comparative evidence and clinical positioning of TNF-α inhibitors in juvenile idiopathic arthritis-associated uveitis.

Agent/route	Evidence base	Reported ocular outcomes	Clinical positioning and limitations
ADA, SC	SYCAMORE randomized trial: 90 children with active JIA-U despite MTX (12); supported by systematic reviews and cohorts (13–18)	Treatment failure: 16/60 (27%) with ADA + MTX vs. 18/30 (60%) with placebo + MTX; HR, 0.25 (95% CI, 0.12–0.49; p < 0.0001). Adverse-event rate: 10.07 vs. 6.51 events per patient-year.	Best-supported monoclonal TNF inhibitor and preferred after inadequate MTX response. Secondary loss of response may prompt TDM, dose intensification, or switching.
IFX, IV	Pediatric JIA-U and refractory noninfectious uveitis observational studies (20–23)	Frequently controls inflammation, preserves visual acuity, and facilitates glucocorticoid tapering. In one refractory pediatric cohort, 82.4% required doses ≥10 mg/kg (22).	Alternative for severe or recurrent disease or ADA failure; permits supervised dose escalation but requires infusion access and carries infusion-reaction and immunogenicity burdens.
GLM, SC	Retrospective JIA-U cohort after inadequate ADA response: n = 10 (25); broader SC evidence summarized in a systematic review of refractory noninfectious uveitis (26)	All eight patients with secondary ADA loss of response initially responded; neither primary nonresponder responded. Three later lost response; five of 10 remained responders at final follow-up (four complete and one partial).	Convenient outpatient or home administration. Most plausible after secondary or immunogenic ADA failure; ocular use is off-label, and the evidence is low certainty and noncomparative.
GLM, IV	Retrospective JIA-associated anterior uveitis cohort: n = 13 (27)	Eleven of 13 patients (84.6%) responded; anterior chamber activity and flare frequency improved, and cystoid macular edema resolved in all six affected eyes.	Supervised, body-surface-area-based dosing with longer maintenance intervals, but infusion-center resources are required. Evidence is limited to a small retrospective cohort, with no head-to-head comparison with SC GLM.
CZP, SC	Case series of three patients with heterogeneous refractory noninfectious uveitis (29)	All three patients showed clinically meaningful improvement in ocular inflammation, with no treatment-related adverse events reported.	Evidence is indirect for JIA-U, and a JIA-U-specific success rate cannot be estimated. Selected off-label option when better-supported agents are ineffective or unsuitable.
ETN, SC	Long-term JIA registry and comparative safety evidence (30, 31)	Effective for articular disease, but ocular efficacy is inconsistent; new-onset or paradoxical uveitis has been reported.	Not preferred for JIA-U. Regular ophthalmic screening remains necessary during treatment.

Response percentages for SC and IV GLM should not be compared directly because the cohorts were small and retrospective and differed in patient selection, concomitant treatment, follow-up, and response definitions. The systematic review cited for GLM summarized SC regimens and does not directly support IV administration.

### 3.7 Dose optimization and therapeutic drug monitoring

With respect to treatment optimization, a prospective cohort study by Heiligenhaus et al. found that ADA therapy was significantly associated with achieving at least 6 months of uveitis quiescence during follow-up, highlighting its value in maintaining control of ocular inflammation (7). Nevertheless, some patients may have an inadequate response or secondary loss of response to standard biweekly dosing. Dehoorne et al. first proposed a therapeutic window for ADA and suggested an optimal trough concentration range of 9.6–13 μg/mL. Complete responders had a notably higher ADA trough concentration of 11.8 μg/mL, and therapeutic drug monitoring may help identify the cause of an inadequate response and inform dose adjustment or drug switching (32). However, this proposed therapeutic window was derived from a small, single-center retrospective study that included heterogeneous etiologies and anatomic types of uveitis and therefore cannot yet be regarded as an accepted optimal trough range for JIA-U.

An inadequate response to ADA should be further classified as pharmacokinetic or pharmacodynamic failure. The former is commonly associated with poor adherence, weight gain, increased individual clearance, or the formation of AAA and usually manifests as a low trough concentration; the latter refers to persistent intraocular inflammation despite adequate drug exposure. Leinonen et al. included 31 children with JIA-U and detected AAA levels ≥12 AU/mL in nine patients (29%). Higher AAA levels were associated with greater uveitis activity, failure to achieve remission, the absence of concomitant MTX, and lower drug concentrations (33), suggesting that concurrent measurement of adalimumab concentrations and AAA may facilitate management of secondary loss of response. Another study showed that, in some patients, ADA concentrations were already high after treatment intensification, yet the prespecified efficacy criteria were not met, indicating that persistent inflammation is not invariably attributable to inadequate drug exposure (34). In patients with adequate drug exposure but persistent ocular inflammation, simply increasing exposure may offer limited benefit; switching to another monoclonal antibody TNF-α inhibitor or to a therapy with a different mechanism of action should therefore be considered. In a small case series of four patients reported by Burlo et al., articular or ocular inflammation was controlled after patients with an inadequate response to biweekly ADA were switched to weekly dosing, and no apparent adverse events were observed (35). In patients with an inadequate response or secondary loss of response to standard biweekly ADA, weekly dosing may improve inflammatory control in some children and can be considered as a dose-optimization strategy before switching therapy. However, its infection risk, cost, and long-term safety require confirmation in larger studies.

### 3.8 Long-term maintenance, tapering, and discontinuation

**Figure 1:**
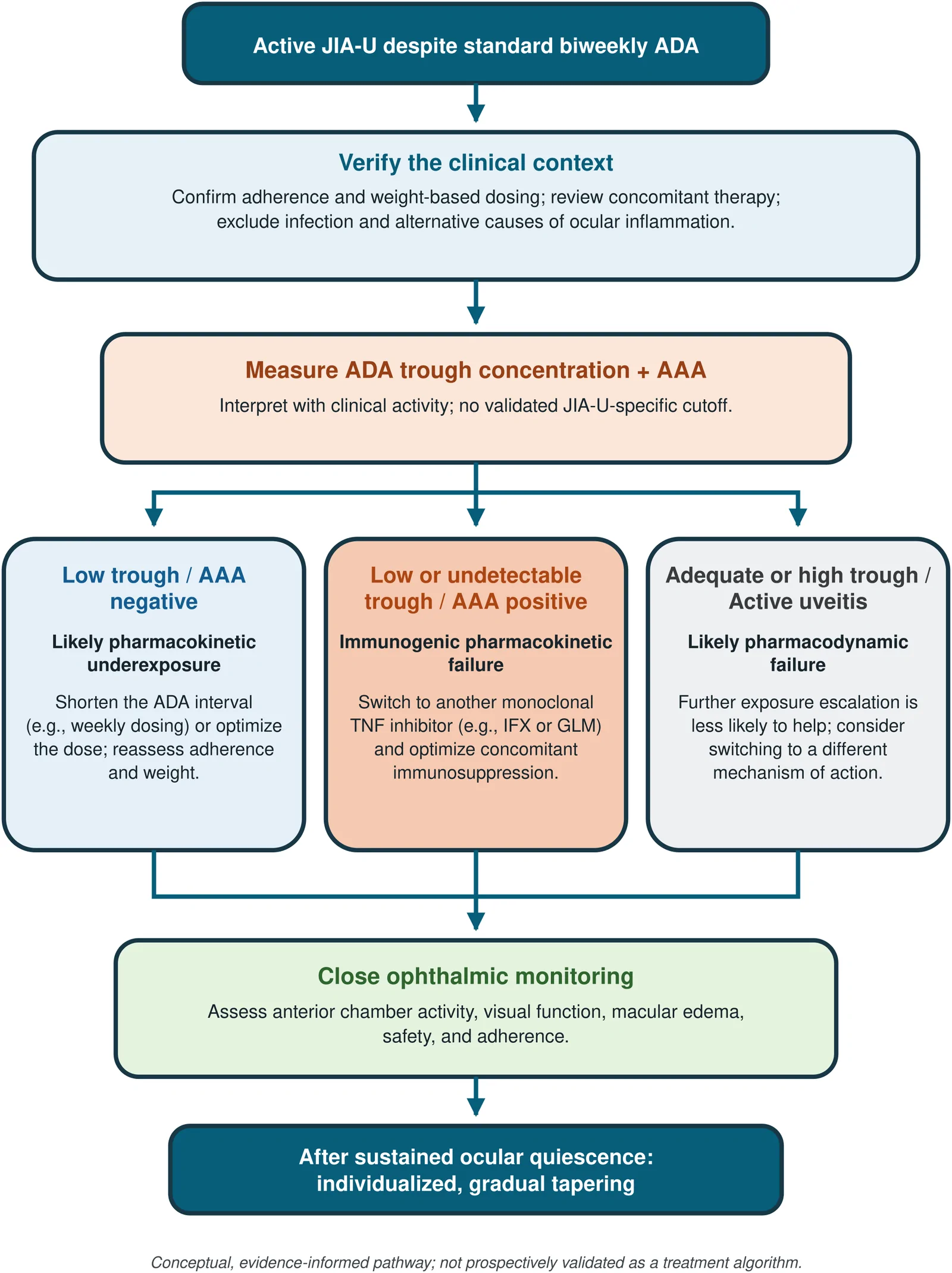
Conceptual treatment algorithm for inadequate response to adalimumab in juvenile idiopathic arthritis-associated uveitis. After confirmation of treatment adherence, weight-adjusted dosing, concomitant therapy, and exclusion of infection or alternative causes of ocular inflammation, adalimumab trough concentrations and anti-adalimumab antibodies may help distinguish pharmacokinetic from pharmacodynamic failure. Treatment decisions should be individualized and accompanied by close ophthalmic monitoring. This algorithm represents a narrative synthesis of the available evidence and has not been prospectively validated. AAA, anti-adalimumab antibodies; ADA, adalimumab; GLM, golimumab; IFX, infliximab; JIA-U, juvenile idiopathic arthritis-associated uveitis; TNF, tumor necrosis factor.

Although ADA has demonstrated clear efficacy in JIA-U, long-term maintenance and the optimal timing of tapering and discontinuation remain key management issues (36). A retrospective case series found that ADA-induced remission of JIA-U generally could not be maintained after treatment discontinuation, whereas overall visual outcomes were favorable during continued therapy (37). This finding suggests that remission during treatment does not necessarily indicate that underlying immune activity has been permanently eliminated. Regarding tapering, a multicenter retrospective cohort study by Marino et al. included 114 children with noninfectious uveitis who underwent ADA tapering, 46% of whom had JIA-U. Uveitis recurred in 46% of patients during tapering, with a median time to recurrence of 30 weeks; gradual extension of the dosing interval was associated with a lower risk of recurrence (38). Previous studies have indicated that ocular inflammatory activity in JIA-U may not parallel the progression of articular disease (17). Decisions regarding tapering or discontinuation should therefore also account for the status of ocular inflammation and the stability of visual function. In patients with stable JIA-U, abrupt discontinuation or rapid tapering of ADA should be avoided in favor of a gradual, individualized strategy. An evidence-informed algorithm integrating therapeutic drug monitoring, treatment adjustment, ophthalmic monitoring, and gradual tapering is presented in **Figure 1**.

### 3.9 Adverse events

Common adverse events associated with TNF-α inhibitors include infections, injection-site reactions, and infusion-related reactions. Serious adverse events are relatively uncommon, but the risks associated with long-term treatment and rare events warrant continued attention. A Cochrane systematic review found that the adverse events reported in randomized trials of JIA-U were generally consistent with the known safety profiles of these agents (16). Safety profiles vary among TNF-α inhibitors. ADA and GLM are associated primarily with subcutaneous injection-site reactions and infections; because of its chimeric antibody structure and intravenous administration, IFX requires particular attention to infusion reactions and immunogenicity. TNF-α inhibitor therapy also warrants vigilance for tuberculosis and other opportunistic infections, together with individualized assessment of the risks of rare demyelinating events, congestive heart failure, and malignancy (39). Overall, TNF-α inhibitors are generally well tolerated, but their safety cannot be assessed solely on the basis of short-term clinical trials. During treatment, monitoring should be tailored to the specific agent and individual patient, with particular attention to infections and administration-related reactions. Long-term follow-up is needed to define the actual risk of rare serious adverse events associated with different agents, doses, and concomitant immunosuppressive regimens.

## 4 Discussion

Conventional glucocorticoids and immunosuppressants remain first-line therapies for JIA-U, but adverse events and inadequate efficacy limit their use in patients requiring long-term maintenance therapy or those with refractory disease. TNF-α inhibitors have broadened the therapeutic options for JIA-U. An inadequate response to ADA may reflect insufficient drug exposure or persistent inflammation despite adequate exposure. After adherence is confirmed, low drug concentrations related to body weight, increased clearance, or anti-adalimumab antibodies (AAA) may support dose intensification or within-class switching. Conversely, persistent disease activity despite adequate exposure may warrant switching to a therapy with a different mechanism of action. Therapeutic drug monitoring should guide, rather than dictate, these decisions. Future research should move beyond efficacy evaluation toward precision treatment, focusing on treatment timing, switching, tapering and discontinuation, and long-term safety. Such efforts will support more comprehensive pharmacotherapeutic management and improve long-term ocular outcomes in children with JIA-U.

### 4.1 Limitations

This review is limited by heterogeneity in diagnostic criteria, screening intervals, ocular grading, outcome definitions, and follow-up across the cited studies. The often asymptomatic onset of pediatric uveitis and discordance between ocular and articular activity may contribute to delayed recognition or misclassification; recent long-term pediatric data further underscore this diagnostic burden (2, 7, 40).

The proposed trough range of 9.6–13 μg/mL was derived from a small, single-center, heterogeneous pediatric uveitis cohort and has not been validated for JIA-U. Prospective studies are needed to establish whether pharmacokinetically guided management improves outcomes over clinically guided escalation.

## 5 Conclusion

ADA is the best-supported, most widely used, and preferred TNF-α inhibitor when conventional immunosuppressive therapy is inadequate. IFX is an important alternative for patients with an inadequate response to ADA. GLM and CZP provide additional options for refractory JIA-U, although the evidence is derived mainly from small studies. ETN is generally not recommended as a preferred treatment for JIA-U. Given the substantial relapse risk after adalimumab tapering or discontinuation and uncertainty surrounding rare long-term adverse events, treatment de-escalation should be gradual and individualized, with close ophthalmic and safety monitoring to balance disease control against cumulative treatment risks and burden.
